# Combining magnetically isolated CD45 cells with serum maintains intact drug responsiveness for ELISpot analysis in clinical trials

**DOI:** 10.1093/immhor/vlae012

**Published:** 2025-01-30

**Authors:** Chris Mavrangelos, Asiri Wijenayaka, Kurt J Sales, Patrick A Hughes

**Affiliations:** Agilex Biolabs, Adelaide, South Australia, Australia; Agilex Biolabs, Adelaide, South Australia, Australia; Adelaide Medical School, University of Adelaide, Adelaide, South Australia, Australia; Agilex Biolabs, Adelaide, South Australia, Australia; Agilex Biolabs, Adelaide, South Australia, Australia; Adelaide Medical School, University of Adelaide, Adelaide, South Australia, Australia

**Keywords:** clinical trial, ELISpot, flow cytometry, magnetic isolation

## Abstract

Enzyme-linked immunosorbent spot analysis is frequently used to investigate immune responsiveness during clinical trials. However, ELISpot classically utilizes peripheral blood mononuclear cell isolates from whole blood, requiring relatively high blood draw volumes and removing both granulocytes and bound drug. Here, we describe a novel protocol whereby CD45 cells are magnetically isolated from human whole blood and co-incubated with serum isolated from the same subject. Infliximab is a well characterized anti-tumor necrosis factor α (TNF-α) antibody in clinical use since the late 1990s. We demonstrated that TNF-α inhibition by infliximab in spiked whole blood is lost on peripheral blood mononuclear cell isolation but remains in serum, and that combining serum from infliximab spiked whole blood with magnetically isolated CD45 immune cells inhibited PMA/ionomycin-stimulated TNF-α secretion. This novel protocol has important implications for enzyme-linked immunosorbent spot analysis in clinical trials in which blood volume is limited, and keeping drug responses intact provides critical information.

## Introduction

Enzyme-linked immunosorbent spot (ELISpot) is a sensitive technique for quantitative analysis of cell secretions and is currently one of the most common methods for pharmacodynamic assessment of cellular immunity in clinical trials.^1^^,^^2^ ELISpot shares principals with enzyme-linked immunosorbent assay (ELISA), whereby cell secreted products including cytokines and immunoglobulins are visualized after antibody capture. However, while ELISA detects targets within a matrix (e.g., serum), ELISpot instead captures targets immediately as they are secreted by cells and is therefore a measure of cell frequency. This rapid capture provides quantification of targets that disappear from matrix due to lability, bystander cell internalization, and/or binding to soluble or extracellular receptors. First developed in the early 1980s to detect antibody-secreting cells^3^^,^^4^ and bacterial endotoxin,^5^ ELISpot analysis of clinical and preclinical tissues has since provided fundamental insight into the development of vaccines and mechanisms underlying disease states including but not limited to cancer, allergy, transplantation, and HIV.^6–8^

Rapid expansion and improvement of components including antibodies and detection reagents have facilitated the utility of ELISpot, as with other techniques; however, the underlying protocol remains relatively unchanged over the last 3 decades. Human ELISpot studies overwhelmingly use peripheral blood mononuclear cell (PBMC) isolates as the cell type of choice, particularly when investigating T cells, and therefore PBMC quality is critical.^2^^,^^9^ The benefits of using PBMC for ELISpot include relative ease of isolation, inexpensiveness, improved in signal-to-noise ratio, high responsiveness to stimulators, and amenability for cryostorage. However, PBMC use also has limitations including relatively high blood volume requirements, induction of changes in biology, and removal of potential environmental cues such as dosed drug, each of which may alter immune responsiveness to stimulation. These limitations have important implications for the analysis of pharmacodynamic effects in clinical trials.

Analysis of purified specific immune cell populations that are isolated by flow cytometric cell sorting or by magnetic isolation have also been applied to ELISpot. However, while well-developed protocols for isolating antibody secreting cells from whole blood for ELISpot analysis exist,^10^ the other major immune cell populations are typically isolated from PBMC and therefore experience the same potential issues as outlined previously. Recent advances in magnetic technology now permit isolation of specific immune populations from whole blood rather than PBMC without the requirement for a cell sorting flow cytometer. These improvements may address some of the limitations of working with PBMC, as blood volume requirements are reduced; however, the application of this technology to ELISpot is yet to be assessed. Serum is almost always used as a blocking step in the initial ELISpot stages, and there is extensive literature on the use of serum for this purpose.^1^ However, there is little information regarding the use of drug-spiked serum for ELISpot.

Here, we spiked whole blood with infliximab, a well characterized anti-tumor necrosis factor α (TNF-α) chimeric antibody used to treat autoimmune diseases including inflammatory bowel disease, arthritis, ankylosing spondylitis, and psoriasis. Infliximab is well known to partition into serum with a reported peak serum concentration (C_MAX_) of approximately 120 µg/mL.^11^ We aimed to assess whether serum containing infliximab at a concentration <50% of C_MAX_ inhibits TNF-α secretion in stimulated lymphocytes isolated from whole blood. We demonstrate that magnetically isolated CD45 cells are suitable for ELISpot analysis and that drug response remains intact when these cells are combined with serum, providing proof of concept for use in longitudinal clinical trials in which blood volume may be limited and pharmacodynamic assessment requires drug response to be intact.

## Materials and methods

### Ethics

Whole blood was obtained from 10 age- and sex-matched volunteer donors (40% male, age 32.3 ± 8.4 years [female] vs 40.5 ± 12.4 years [male]). Informed consent was obtained from all volunteers after the nature and possible consequences of blood donation had been fully explained to them. This study was approved by the Bellberry Limited ethics committee.

### PBMC and serum isolation

PBMC were isolated as previously described.^12^^,^^13^ Briefly, 9 mL of whole blood in a NaHep vacutainer (BD) was overlayered onto Ficoll-Hypaque (GE), centrifuged, and the buffy coat collected. The buffy coat was then washed with Dulbecco’s phosphate-buffered saline (PBS), and viability and cell count were determined by trypan exclusion (Countess; Thermo Fisher Scientific). PBMC all demonstrated viability >80% and were frozen at 1 × 10^7^ cells/mL in freezing media consisting of 15% dimethyl sulfoxide with 25% heat-inactivated fetal bovine serum (FBS) (Thermo Fisher Scientific). Serum was collected from whole blood in a clot activator vacutainer (BD) serum tube after centrifugation and stored at −20 °C until use. Some whole blood was spiked with the clinically relevant concentration of the anti-TNF-α antibody 50 µg/mL infliximab (Sigma-Aldrich)^11^ and incubated at room temperature for 1 h prior to PBMC or serum isolation.

### ELISpot

Frozen PBMC were thawed and washed, and viability and cell count were determined by trypan exclusion as previous, and all PBMC had viability >80%. Thawed PBMC or magnetic-activated cell sorting (MACS)–isolated cells were rested overnight at 37 °C in 5% CO_2_ before being resuspended to 1 × 10^6^ cells/mL in RF-10 complete media (10% heat-inactivated FBS, 100 U/mL penicillin/streptomycin, and 1x GlutaMAX in RPMI 1640; all from Thermo Fisher Scientific) and plated at a density of 2 × 10^4^ live cells/well in RF-10 blocked TNF-α ELISpot plates (Mabtech) in the presence or absence of 50 ng/mL PMA (Sigma-Aldrich) and 1 µg/mL ionomycin (Sigma-Aldrich), known to stimulate TNF-α secretion,^13^ and/or 5% serum in RF-10 media for 16 h. ELISpot development was then performed essentially as per the manufacturer’s recommendations. Briefly, 0.5 µg/mL α-TNF-biotin in 0.5% FBS in PBS was added to each well and incubated at room temperature for 2 h prior to addition of 1:1,000 diluted Strepavidin-ALP and incubation for 1 h at room temperature followed by addition of 0.5% FBS in PBS 0.45 µm filtered BCIP/NBT-plus for 20 min before the reaction was stopped by flooding the wells with MQ water. Plates were then dried and analyzed on an iSPOT Fluorospot reader (AID GmbH) under standard conditions.

### CD45 cell isolation from whole blood

CD45+ cells were isolated from whole blood using CD45 microbeads essentially according to the manufacturer’s recommendations. Briefly, 2 mL of whole blood was labeled with 100 µL StraightFrom CD45 MicroBeads (Miltenyi Biotec) and incubated for 15 min at 4 °C. The labeled blood was then loaded onto a whole blood column (Miltenyi Biotec) placed in a MACS separator magnet (Miltenyi Biotec) and washed, and labeled cells were eluted with elution buffer.

## Statistics

All data are presented as mean ± SD. Paired *t* test or 1-way analysis of variance with repeated measures and Tukey’s post hoc tests determined the significance of data as indicated.

## Results

### TNF-α–inhibiting effects of infliximab are lost on PBMC isolation but retained in serum

**Figure 1.**
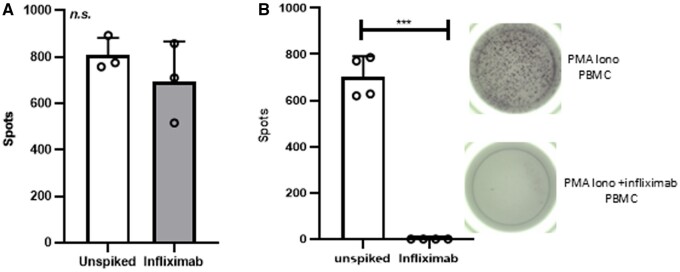
The effect of infliximab on TNF-α is lost in PBMC isolation but kept in serum. (A) PBMC were isolated from whole blood that had been spiked with infliximab at 50 µg/mL and incubated for 1 h at ambient temperature (filled bars) or not (unfilled bars). (B) PBMC were isolated from whole blood and plated in the presence of PMA/ionomycin with 5% serum made from whole blood that had been spiked with or without Infliximab. n = 3. Mean ± SD; paired *t* test. ***P < 0.001. n.s., not significant.

We initially demonstrated that PBMC are not suitable for investigating the effects of drug on cytokine secretion by ELISpot. Spiking whole blood with 50 µg/mL infliximab prior to isolating PBMC did not inhibit the number of TNF-α spots induced by PMA/ionomycin stimulation, when compared with PBMC isolated from whole blood that had not been spiked (n = 3; 809 ± 74 spots [not spiked] vs 695 ± 172 spots [infliximab spiked]) (Fig. 1A).

We also observed that the addition of 5% serum in RF-10 media did not alter PMA/ionomycin-stimulated TNF-α production from PBMC, but higher serum concentrations did (data not shown). However, 5% serum from 50 µg/mL infliximab-spiked whole blood completely inhibited PMA/ionomycin-stimulated TNF-α production from PBMC when compared with serum from unspiked whole blood (702 ± 89 [unspiked serum] vs 1 ± 1 spots [5% infliximab-spiked serum]; *P* < 0.001; n = 4) (Fig. 1B). These results indicate that infliximab does not partition into PBMC isolations, but it does partition into serum, and the addition of serum from drug-treated subjects is useful for ELISpot analysis in clinical trials.

### Serum from infliximab-spiked whole blood inhibits TNF-α secretion from magnetically isolated CD45+ cells from whole blood

**Figure 2.**
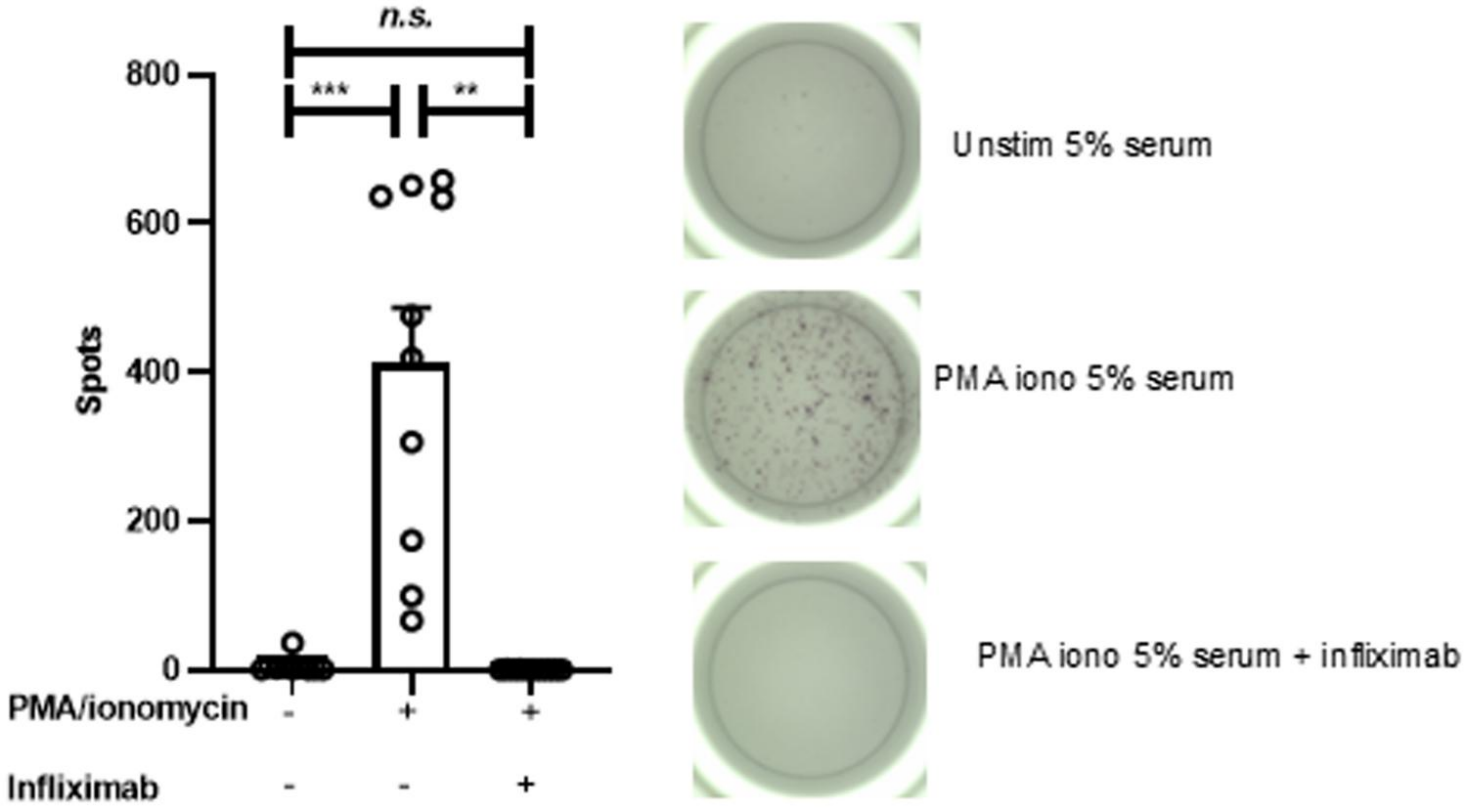
Serum from infliximab-spiked whole blood inhibits TNF-α secretion from magnetically isolated CD45+ cells from whole blood. TNF-α secretion from magnetically isolated CD45+ immune cells from whole blood was inhibited by serum from whole blood that had been spiked with infliximab. n = 10. Mean ± SD; 1-way repeated measures analysis of variance with Tukey’s post hoc test. **P < 0.01, ***P < 0.001. n.s., not significant.

We next demonstrated that PMA/ionomycin-stimulated TNF-α secretion from CD45 MACS-isolated cells from whole blood is almost completely inhibited in the presence of 5% serum isolated from whole blood samples that had been spiked with infliximab from the same donor (n = 10; 6 ± 11 [unstimulated] vs 412 ± 237 [PMA/ionomycin stimulated] vs 1 ± 1 [PMA/ionomycin stimulated in the presence of infliximab]) (Fig. 2). These findings demonstrate that CD45 MACS isolated cells are suitable for ELISpot analysis, and that, when combined with serum, drug effect remains intact.

## Discussion

PBMC are overwhelmingly used as matrix for ELISpot assays; however, the immune subset proportions differ between PBMC and whole blood, and dosed drug is typically lost during PBMC isolations. Here, we revealed an update to the traditional ELISpot assay by demonstrating that drug responsiveness can be maintained and draw volume decreased by combining serum from whole blood with magnetically isolated CD45+ cells. This updated assay more closely reflects dosed subject samples in clinical trials than standard PBMC isolations.

PBMC isolations by density centrifugation were first described over 4 decades ago and, in general, the same principals apply. Mononuclear cells, including T cell, B cell, natural killer cell, and monocyte populations, are separated away from denser erythrocytes, platelets, and cells with multilobed nuclei including neutrophils and eosinophils. PBMC have been the matrix of choice for ELISpot assay for decades. There are several benefits of PBMC that apply not only to ELISpot, but also to associated techniques including ELISA analysis and flow cytometry. First, PBMC isolations are relatively simple in terms of technical ability, equipment, and expense. More recent advances have further eased PBMC isolation, including specialized vacutainers where PBMC can be isolated straight from blood draw in a laboratory with standard equipment. Second, PBMC are a relatively simple matrix compared with whole blood, improving signal-to-noise detection that can limit multiple technologies including ELISpot. Third, thawed PBMC are in a resting state and therefore highly responsive to stimulators. Furthermore, as they contain a relatively high proportion of T cells, stimulation with specific T cell stimulators and peptide pools that discriminate between major histocompatibility complex class I and II binding are commonly used in ELISpot assays.^14^ Finally, when prepared correctly, PBMC are amenable to long-term storage of several years in liquid nitrogen. This is particularly relevant to longitudinal clinical trials in which analysis can be batched, limiting the potential of technical variability that may arise due to different operators and/or equipment in downstream assays.

It is important to note that the use of PBMC is not without limitations. A relatively high blood volume is required for PBMC isolation, and in the previous experiments we isolated PBMC from 9 mL of whole blood compared with 2 mL used for magnetic isolation of CD45+ cells. Relatively high volumes of whole blood are typically required for PBMC isolation as (1) isolation from low blood volumes can be technically challenging and (2) PBMC are typically frozen in specialized media at not <0.5 × 10^6^ cells, and numbers less than this have been demonstrated to be suboptimal.^15^ While ELISpot does not require the high number of PBMC produced from 9 mL of blood, and these cells may be used for other techniques in combination with ELISpot, thawed PBMC that have previously been cryopreserved do not maintain viability when refrozen. This volume of blood is high compared with that used in flow cytometry or serum production. This requirement for high volume may impede PBMC production during longitudinal clinical trials, particularly when draws are required at multiple time points in the multiple ascending dose phase, or in populations in which blood volume is limited, for example in pediatric populations. Furthermore, the isolation and cryogenic storage of PBMC is known to alter cellular biology relative to steady-state whole blood. For example, thawed PBMC revert to a resting state, with downregulation of markers known to be involved in activation and proliferation including CD62L, Ki67, and CD69.^16^^,^^17^ Changes in activation status in particular warrant the necessity to stimulate PBMC for cytokine excretion for ELISpot analysis, and therefore the aim of the experiment changes from asking “How does a condition alter mediator secretion?” to “How does a condition alter responsiveness to stimulation driving mediator secretion?” This limitation may have important implications if ELISpot is used in isolation but can easily be overcome when it is combined with other techniques such as flow cytometry and ELISA. Finally, and importantly for pharmacodynamics assessment during clinical trials, there is a high risk that dosed drug is removed from the PBMC isolate during the procedure. We demonstrate this in the previous study, as the TNF-α–inhibiting effect of infliximab was lost in PBMC isolated from infliximab-spiked whole blood. As previous, this potentially has implications in which ELISpot analysis of PBMC is extrapolated to inform clinically relevant outcomes.

The ability to purify specific immune cell populations for downstream analysis using proteomic and genetic technologies has provided a wealth of information regarding the mechanisms underpinning immunology in health and disease.^18^ These initial isolations were performed using fluorescence-activated cell sorting, with isolation using magnetic beads following soon after in the late 1990s.^19^ Magnet-based isolations are widely regarded as easier than fluorescence-activated cell sorting, as they require less complicated equipment and technical expertise. As an example, in the previous method, we isolated CD45+ cells in under 30 min. Recent developments in bead-based technologies include the development of reagents that can isolate immune cells from whole blood. CD45 is a protein tyrosine phosphatase receptor type C, and was originally termed “leukocyte common antigen” given its widespread expression on all differentiated hematopoietic cells except erythrocytes and platelets, and not on other cell types including stromal cells.^20^ The composition of CD45+ cells in whole blood and PBMC is known to differ due to a lack of granulocytes in PBMC. Granulocytes are also isolated in the magnetic isolation of CD45+ cells from whole blood, implying that ELISpot of cells that are magnetically isolated from whole blood shares closer clinical relevance to whole blood than PBMC. Furthermore, we also demonstrate that enough cells are magnetically isolated from a 2 mL sample of blood to perform ELISpot analysis, a substantial reduction in blood volume compared with PBMC and therefore more suited to longitudinal analysis in clinical trials.

Loss of drug from matrix during the PBMC isolation process is an important limitation associated with the use of PBMC for pharmacodynamic analysis in clinical trials, as the impact on immune cell biology may affect outputs from functional assays such as ELISpot. We demonstrated that the drug effect can be replicated using serum from whole blood investigating either PBMC or magnetically isolated CD45 cells. Serum from fetal calf or bovine sources has traditionally been used in ELISpot and other assays to block background signals. Here, we replaced serum from these sources with serum from whole blood isolates from humans. Infliximab was an ideal drug candidate to test in these conditions, as it is known to partition into serum and potently binds TNF-α, an established ELISpot target. Infliximab is efficacious in Crohn’s disease treatment in which C_MAX_ is approximately 120 µg/mL,^11^ so we tested a spiking concentration of 50 µg/mL, as it is <50% of C_MAX_. We observed that serum concentrations had to be diluted to 5%, as higher concentrations resulted in blocking signal with both infliximab-spiked and nonspiked serum. Therefore, the resulting effective infliximab concentration in the ELISpot wells was 2.5 µg/mL, indicative of a sensitive assay. Furthermore, serum can easily be obtained from a low whole blood volume vacutainer, again reducing the amount of whole blood required to be drawn from subjects in clinical trials.

## Conclusion and future perspectives

The current study provides proof of concept for using magnetic isolation of lymphocytes from whole blood for ELISpot analysis and also demonstrates the utility of serum from dosed subjects in ELISpot assays. Further repeat testing across multiple drugs, preferably alongside traditional ELISpot, is required to demonstrate robustness for incorporation into clinical trials. Of interest would be to test whether these results extend not only to drugs that target soluble mediators, but also to cell surface receptors and second messenger systems, and to refine the immune population to more specific subtypes. Here, we isolated serum and CD45+ cells from separate tubes obtained at the same blood draw. This combined total blood draw is lower than that typically required for PBMC, reducing the total blood draw required per time point in a longitudinal clinical trial. Furthermore, while the cryopreservation conditions for lymphocytes isolated by magnetic beads are likely similar to those for PBMC, suitable conditions for cryopreserving magnetically isolated granulocytes have not been established, limiting the utility of this technique for their batch analysis. This protocol combines relatively quick and simple additions to the ELISpot workflow without major cost implications and are therefore easily adopted. The combination of magnetically isolated cells from whole blood with subject serum adds to the clinical relevance of ELISpot with potentially far-reaching consequences.

## Data Availability

The data underlying this article are available in the article and in its online supplementary material.
